# In knee osteoarthritis, the production of cytokines and metalloproteinases in presence of chondrocytes and CD4^+^ T cells depends on T cell subset: An in vitro analysis

**DOI:** 10.1016/j.ocarto.2025.100642

**Published:** 2025-06-18

**Authors:** H. Platzer, M. Wellbrock, G. Pourbozorg, R. Mayakrishnan, S. Gantz, B. Khamees, S. Maciej, B. Moradi

**Affiliations:** a Orthopedic Research Center, Kiel University, 24118 Kiel, Germany; b Department of Orthopedics and Trauma Surgery, University Medical Center Schleswig-Holstein, Campus Kiel, 24105 Kiel, Germany

**Keywords:** Osteoarthritis, T cells, Chondrocytes, Cytokines, Metalloproteinases, Inflammation

## Abstract

**Objective:**

Osteoarthritis (OA) is driven by biomechanical and biochemical inflammatory processes, including CD4^+^ T cell infiltration and activation. However, the role of CD4^+^ T cell subsets interacting with neighboring cells shaping the local inflammatory milieu have remained largely unexplored. This study aimed to investigate in vitro whether interaction of chondrocyte and CD4^+^ T cells modulate cytokine and metalloproteinase production in OA, and to determine if this modulation differ depending on CD4^+^ T cell subsets.

**Method:**

Nineteen patients with knee OA undergoing knee replacement were enrolled. From peripheral blood CD4^+^ T cells were isolated and differentiated into subsets (Th1, Th2, Th17, Treg) using a novel developed protocol. T cell differentiation was validated by flow cytometry. Chondrocytes were mono- and co-cultured with T cell subsets and in culture supernatant cytokine and metalloproteinase levels were quantified using ELISA and multiplex assays.

**Results:**

Compared to monocultures levels MMP-1/3/9/13 and IL-6 were elevated in all co-cultures of chondrocytes and CD4^+^ T cell subsets, with the highest levels in Th17 co-cultures. GM-CSF, IL-9, IL-17 were specifically elevated in Th17 co-cultures and IFN-γ in Th1 co-cultures. TNF-α production was significantly reduced only in Treg co-culture compared to monoculture approach.

**Conclusion:**

This study indicates that chondrocytes can interact with CD4^+^ T cell subsets in OA, modulating the production of metalloproteinases and cytokines to varying degrees, depending on the CD4^+^ T cell subset. Our findings can open new avenues in OA treatment using T cell-based or T cell subset-targeted therapies to modulate inflammatory patterns in affected OA joints.

## 1 Introduction

Osteoarthritis (OA) pathology is complex, resulting from biomechanical and biochemical inflammatory interactions. An increased presence of cartilage-degrading enzymes and an imbalance of pro- and anti-inflammatory cytokines in osteoarthritic joints contribute to progressive cartilage breakdown [1]. Elevated pro-inflammatory cytokine levels, such as tumor necrosis factor-alpha (TNF-α) and interleukin-6 (IL-6), are found in synovial fluid, cartilage, and subchondral bone of OA patients [2]. These cytokines foster an inflammatory environment driving joint catabolism and promoting cartilage-degrading enzyme synthesis [[2], [3], [4]]. Proteolytic enzymes involved in cartilage breakdown in OA include “a disintegrin and metalloproteinase with thrombospondin motifs” (ADAMTS)-4, −5, and −7 and Matrix-metalloproteinases (MMP)-1, −3, −9, and −13 [[5], [6], [7], [8]]. These enzymes cleave collagen triple helices and degrade non-collagenous matrix components, playing a central role in cartilage degradation and OA pathogenesis [6]. The primary sources of these enzymes and cytokines in OA are cells from synovial tissue and chondrocytes [4,6,8,9]. However, the triggers that lead to a release of this catabolic mechanisms are still unknown.

Chondrocytes adopt a "dedifferentiated-like" phenotype in OA, marked by increased catabolic activity and heightened production of proteolytic enzymes and pro-inflammatory cytokines [10,11]. Hereby chondrocytes can respond to damage-associated molecular patterns via innate immune receptors [11] and can act as antigen-presenting cells, suggesting a role in adaptive immunity through cartilage antigen presentation [11]. Although the absolute infiltration of CD4^+^ T cells in OA joints remains low compared to rheumatoid arthritis joints [12], they are still among the most abundant immune cells infiltrating OA joints [[13], [14], [15]]. CD4^+^ T cells can be divided into different subsets. While T helper (Th)1 and Th17 ​cells are known for their inflammatory properties, Tregs and Th2 cells attributed anti-inflammatory potential [9,16,17]. In OA joints CD4^+^ T cells become polarized into their pro-inflammatory subsets [1,13,18] and infiltrate not only synovial tissue but have also been detected in OA joint space [[18], [19], [20]]. Hence, CD4^+^ T cell subsets might interact directly with chondrocytes in context of OA. CD3^+^ T cells have been shown the ability to interact with OA chondrocytes in vitro, altering metalloproteinase production [21]. Additionally, Th1 and Th17 cytokines, such as Interferon-gamma (IFN-γ) and IL-17, have been shown to induce chondrocyte gene expression linked to inflammation and cartilage degradation [22]. These results indicate a CD4^+^ T cell subset specific impact on chondrocytes and its inflammatory potential, which has not been investigated in OA so far. Understanding this could reveal whether modifying CD4^+^ T cell differentiation or targeting specific subsets could prevent cartilage breakdown and treat OA.

To our knowledge, this is the first study investigating whether CD4^+^ T cell subsets alter the metalloproteinase and cytokine release in presence of chondrocytes from knee OA patients. We developed a novel CD4^+^ T cell polarization assay and an in vitro co-culture system of chondrocytes from OA patients and CD4^+^ T cell subsets, including Th1, Th2, Th17, and Tregs. T cell polarization and subset stability during culture were confirmed by flow cytometry analysis using intra- and extracellular markers. Subsequently, we analyzed cytokine and MMP concentrations in chondrocyte and T cell subset mono- and co-culture supernatants.

## 2 Material and methods

### 2.1 Study population

19 patients (12 women, 7 men) undergoing knee arthroplasty were included in this study. The study population characteristics are summarized in Table 1. We determined osteoarthritis at University Hospital Schleswig-Holstein, Campus Kiel based on patient history, clinical examination, and radiographic evaluation according to the American College of Rheumatology criteria. All patients had osteoarthritis with Kellgren–Lawrence (K&L) scores III or IV. None of the patients had a history of underlying inflammatory disease, intake of DMARDs, intra-articular injection of corticosteroids, hyaluronic acid, or regular intake of an NSAID within three months before surgery. Preoperative laboratory results such as C-reactive protein (CRP) and white blood cells (WBC) were within the normal range for all patients. This study was approved by the Ethics Committee of the University of Kiel (approval code: D405/22), and all patients provided written informed consent prior to study enrollment.

**Table 1:** Demographic and clinical parameters of the study population. Data are presented as mean ​± ​standard deviation, as number (n) or as percentage (%). BMI ​= ​body mass index; IQR ​= ​interquartile range; K&L score ​= ​Kellgren and Lawrence score; UKA ​= ​unicompartmental knee arthroplasty; TKA ​= ​total knee arthroplasty.

	Total study population	Multiplex Analysis (Cytokines)
Number of patients, n	19	9
Age at surgery, years		
Mean ​± ​SD (IQR)	62.8 ​± ​13.5 (21.0)	61.6 ​± ​16.6 (30.0)
Gender, n (%)		
Female	12 (63 ​%)	5 (56 ​%)
Male	7 (37 ​%)	4 (44)
BMI (kg/m^2^)		
Mean ​± ​SD (IQR)	29.0 ​± ​5.6 (9.0)	30.4 ​± ​5.2 (4.2)
K&L score, n (%)		
3	13 (68 ​%)	5 (56 ​%)
4	6 (32 ​%)	4 (44 ​%)
Operation Side, n (%)		
Right	9 (47 ​%)	4 (44 ​%)
Left	10 (53 ​%)	5 (56 ​%)
Operation Type, n (%)		
UKA	17 (89 ​%)	9 (100 ​%)
TKA	2 (11 ​%)	0 (0 ​%)
Leucocytes/nL		
Mean ​± ​SD (IQR)	6.7 ​± ​1.8 (2.4)	6.7 ​± ​1.7 (1.9)
C-reactive protein (mg/L)		
Mean ​± ​SD (IQR)	2.5 ​± ​2.0 (3.0)	1.7 ​± ​2.0 (1.9)

### 2.2 Sample collection

Articular cartilage of the tibial plateau was harvested during surgery and further processed under sterile conditions. 20–30 ​mL ethylenediaminetetraacetic acid (EDTA)-anticoagulated blood was collected immediately before surgery.

### 2.3 Cell preparation and isolation

Peripheral blood mononuclear cells (PBMCs) were isolated from peripheral blood (PB) using Ficoll-Paque™ PLUS (Cytiva, GE Healthcare, Chicago, IL, USA) density gradient centrifugation. An aliquot of PBMCs was reserved for flow cytometry staining. The remaining cells underwent further processing for isolation of CD4^+^ T cells using the Naive CD4^+^ T Cell Isolation Kit II. We developed a novel protocol suiting our study design to polarize naïve CD4^+^ T cells into Th1, Th2, Th17, and Treg cells. Therefore, CD4^+^ T cells were activated using the T Cell Activation/Expansion Kit and T Cell Transact and polarized into different Th subsets by adding the following cytokines based on described CD4^+^ T cell differentiation processes [9,23,24]: IL-12 and anti-IL-4 for Th1 differentiation; IL-4, anti–IFN–γ, and anti-IL-12 for Th2 differentiation; IL-1β, TGF-β, IL-6, IL-23, anti–IFN–γ and anti-IL-4 for Th17 differentiation; TGF-β, rapamycin, and IL-2 for Treg differentiation. CD4^+^ T cell subset populations were then cultured for five days in 96 well plates using TexMACS culture medium supplemented with penicillin/streptomycin (Thermo Fisher Scientific, Inc.) and IL-2. On day 6, cells were harvested, washed, and counted. A small sample was analyzed by flow cytometry to confirm successful polarization, and the remaining cells were primed for subsequent mono- and co-cultivation with chondrocytes, which were being simultaneously processed. All kits, cytokines and antibodies were sourced from Miltenyi Biotec (Bergisch Gladbach, Germany) if not stated otherwise.

Chondrocytes were isolated as described before [25]. Autologous tibial plateau resections were washed with Phosphate-Buffered Saline (PBS). The cartilage was dissected from the underlying bone with sterile scalpels and minced finely. 1 ​× ​10^6^ ​cells were cultured in 25 ​mL of Dulbecco’s Modified Eagle Medium (DMEM), supplemented with 10 ​% fetal calf serum (FCS) and 1 ​% penicillin/streptomycin, in a 5 ​% CO_2_ incubator at 37 ​°C for five days. The culture medium was refreshed two days post-surgery. Five days post-surgery, the cells were detached using trypsin-EDTA, washed with PBS, and resuspended in RPMI-1640 medium, supplemented with 10 ​% FCS and 1 ​% penicillin/streptomycin, at a concentration of 1 ​× ​10^6^ ​cells/mL.

### 2.4 Chondrocytes monoculture and Co-culture with CD4^+^ T cell subsets

Chondrocytes and polarized T cells were cultured under the same conditions in mono- and co-cultures. 5 ​× ​10^4^ chondrocytes and/or 1 ​× ​10^5^ ​Th cells were cultured in 24-well plates at 37 ​°C for 48 ​h. Supernatants were collected and stored at −80 ​°C until further use. Cell numbers were chosen based on prior experiments [21,25,26].

### 2.5 Flow cytometry analysis

CD4^+^ T cell subsets (Th1, Th2, Th17, and Treg) were identified by multicolor flow cytometry before and after T cell polarization (extracellular N ​= ​19; intracellular N ​= ​3) and following cell mono- (N ​= ​6) and co-culture (N ​= ​3), to verify successful differentiation and confirm their phenotype maintenance during experiment. Antibodies and cell preparation solutions utilized in this study were purchased from Miltenyi Biotec (Bergisch Gladbach, Germany) unless stated otherwise. T cells were first incubated with a fixable live/dead dye (LIVE/DEAD™ Fixable Near IR (780) Viability Kit, Thermo Fisher Scientific). Cells were extracellularly stained with fluorescently-coupled antibodies against CD3 (REA613-VioGreen), CD4 (REA623-VioBrightR720), C–C Chemokine Receptor (CCR)10 (REA326-PE), CCR4 (REA279-APC), C-X-C-Chemokin-Rezeptor (CXCR)3 (REA232-PE/Vio770), CCR6 (REA190-PE/Vio615), CD25 (REA570-VioBright), and CD127 (REA614-PE/Vio770). For intracellular staining, cells were fixed and permeabilized with forkhead-Box-Protein P3 (FoxP3) staining buffer and incubated with antibodies against RAR-related orphan receptor gamma (RORyt) (REA278-VioBright515), GATA binding protein 3 (GATA-3) (REA174-PE), T-box-expressed in t-cells (T-bet) (REA102-APC), Helios (REA829-FITC), and FoxP3 (REA1253-APC). Flow cytometric analysis was performed using a MACSQuant16 Analyzer (Miltenyi Biotec, Bergisch Gladbach, Germany). Data were analyzed using FlowJo, version 10.8.1 (FlowJo, Ashland, OR, USA).

### 2.6 ELISA and multiplex assay

Enzyme-linked immunosorbent assays (ELISAs) from Antibodies.com Europe AB (Stockholm, Sweden) were utilized to measure enzyme concentration of MMP-1 (Catalog No. A2813), MMP-3 (Catalog No. A665), MMP-9 (Catalog No. A668), MMP-13 (Catalog No. A1488), ADAMTS-4 (Catalog No. A74633), ADAMTS-5 (Catalog No. A74634), and ADAMTS-7 (Catalog No. A3760) in chondrocyte and CD4^+^ T cell subset mono- and co-culture supernatants, according to manufacturer's instructions. Photometric analysis used a SpectraMaxID3 (Molecular Devices, San Jose, CA, USA).

MACSPlex Cytokine 12 kit assays (Miltenyi Biotec, Bergisch Gladbach, Germany) measured cytokines Granulocyte-macrophage colony-stimulating factor (GM-CSF), IFN-γ, IL-2, IL-4, IL-5, IL-6, IL-9, IL-10, IL-12, IL-17A, and TNF-α in culture supernatants. Supernatants were analyzed per manufacturer's instructions using MACSQuant16 with MACSQuantify software (Version 2.13.3; Miltenyi Biotec, Bergisch Gladbach, Germany).

### 2.7 Statistical analysis

Continuous variables were presented as means ​± ​standard deviations, and discrete variables as counts and percentages. Shapiro-Wilk test confirmed non-normality for most datasets. Non-parametric tests were chosen to enhance robustness and decrease outlier effects. For cytokine and enzyme analyses, Friedman and Wilcoxon matched-pair signed-rank tests were used with Bonferroni adjustment. Analyses were two-tailed conducted using SPSS (IBM SPSS Statistics, Version 27.0 Armonk, NY, USA) and figures created with GraphPad Prism V10 (GraphPad Software, San Diego, CA, USA).

## 3 Results

### 3.1 Polarization of CD4^+^ T cell subsets

**Fig. 1:**
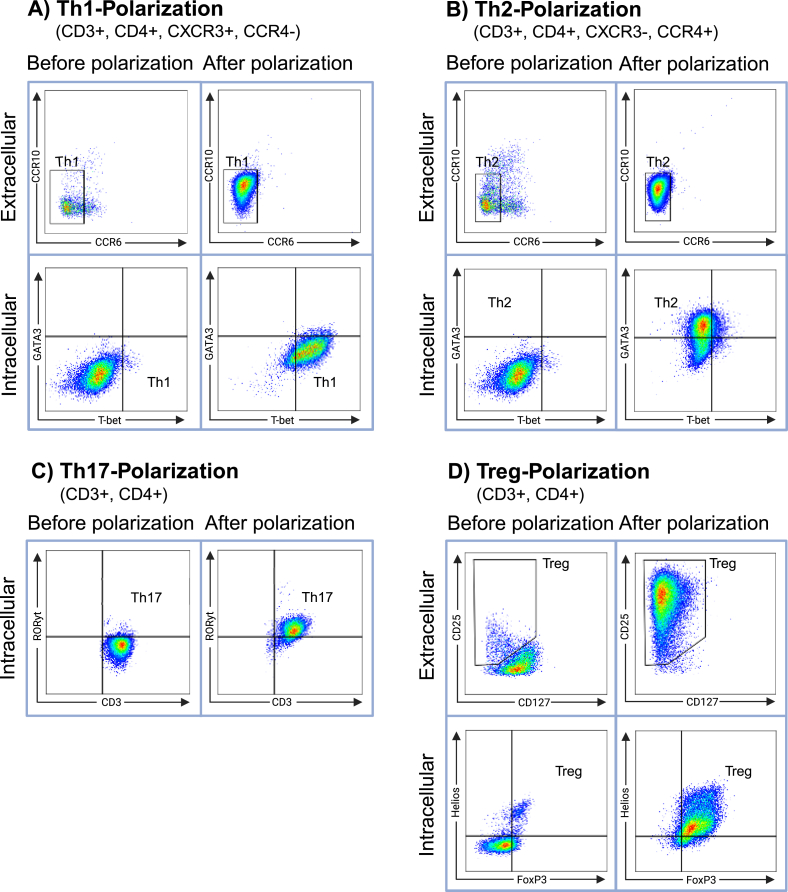
**T cell subsets before and after respective polarization process assessed by multi-color flow cytometry**. T cell subsets were analyzed by flow cytometry before and after polarization. Successful polarizations of CD3^+^CD4^+^CXCR3^+^CCR6^−^CCR10^−^T-bet^+^ Th1 cells (A), CD3^+^CD4^+^CXCR3^−^CCR4^+^CCR6^−^CCR10^−^GATA3^+^ Th2 cells (B), CD3^+^CD4^+^RORyt^+^ Th17 ​cells (C), and CD3^+^CD4^+^CD25^+^CD127^−^FoxP3^+^ Treg cells (D) are shown by upregulation of T cell subset specific expressing markers post polarization. Each diagram illustrates the endpoint of the gating strategy for Th1 (A), Th2 (B), Th17 (C), and Treg (D) of a representative sample. Lymphocytes were identified according to Forward Scatter (FSC) and Side Scatter (SSC), while doublet cells were excluded using FSC-Area versus FSC-Height parameters. Viable cells were gated based on the exclusion of LIVE/DEAD™ Fixable Near IR 780-positive cells.

**Fig. 2:**
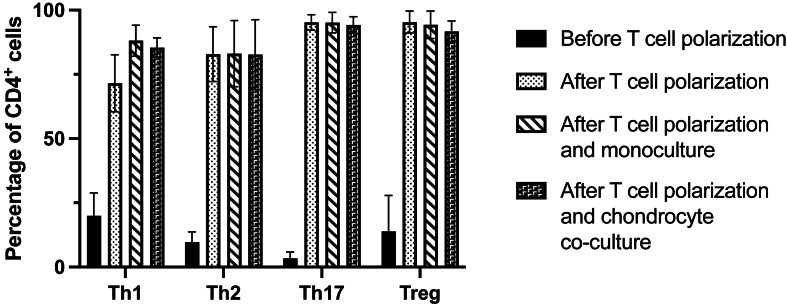
**Differentiation and phenotype maintenance of T cell subsets in mono- and co-cultures with chondrocytes**. CD4^+^ T cells were isolated from PB of OA patients and differentiated into their subsets. Multicolor flow cytometry was performed to prove successful polarization and the maintenance of T cell subsets during cultivation process. Mean and standard deviations of T cell subset (Th1, Th2, Th17, Treg) percentages before polarization, after polarization, after co-culture with chondrocytes and after monoculture are displayed.

T cell subsets were isolated and polarized from PB of 19 patients undergoing knee arthroplasty. We successfully demonstrated the polarization of Th1, Th2, Th17, and Treg subsets with maintenance of these subsets over 48 ​h by extra- and intracellular flow cytometry analysis (representative sample shown in Fig. 1). T cell polarization yielded a significant increase in purity of CD3^+^CD4^+^ T cell population increasing from 37.8 ​± ​11 ​% before polarization to 95.3 ​± ​7.9 ​% after polarization. Substantial increase in CD3^+^CD4^+^CXCR3^+^CCR6^−^CCR10^−^T-bet^+^ cells confirmed successful differentiation of Th1 cells (Fig. 1A). Th2 cells polarization was confirmed by increased population of CD3^+^CD4^+^CXCR3^−^CCR4^+^CCR6^−^CCR10^−^GATA3^+^ cells (Fig. 1B). Post-polarization, the Th2 subset population of CD4^+^ cells increased to 81.1 ​± ​11.7 ​% and remained high after culture (Fig. 2). The proportion of Th17 ​cells expressing RORγt also rose substantially compared to the population prior to polarization, indicating a highly efficient polarization process (Fig. 1D; Fig. 2). Significant enrichment of cells expressing CD25, FoxP3, and Helios, along with low expression of CD127 confirmed successful Treg polarization (Fig. 1D). The proportion of Treg cells increased to 95.3 ​± ​4.2 ​% post polarization (Fig. 2). Upon co-culture with and without chondrocytes, the phenotype of T cell subsets was preserved (Fig. 2), and proliferation and survival rates of Th cells and chondrocytes did not differ significantly when compared using flow cytometry and microscopic cell counts while being cultured under same conditions. The viability of T cells, assessed by flow cytometry, and of chondrocytes, analyzed by trypan blue staining during culture, was above 94 ​% for T cells and above 95 ​% for chondrocytes before and after both mono- and co-cultures.

### 3.2 Enzyme and cytokine concentrations in chondrocyte monocultures

**Fig. 3:**
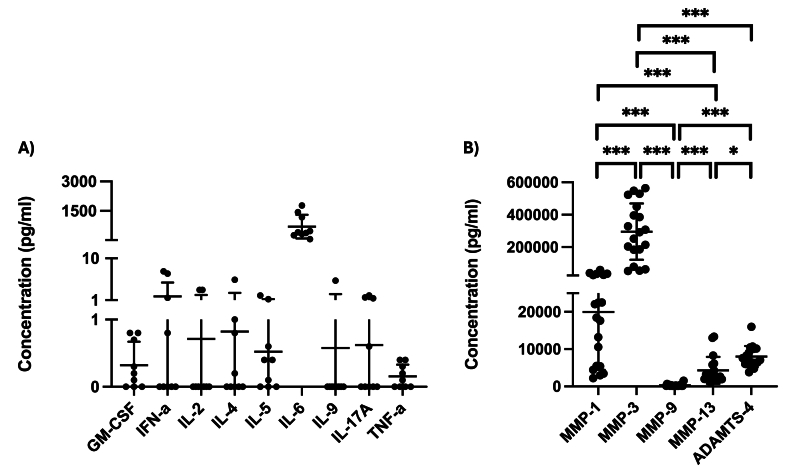
**Production of cytokines and metalloproteinases in chondrocyte monocultures**. Chondrocytes were processed from intraoperatively harvested articular cartilage of the tibial plateau. Cytokine levels in supernatants of chondrocyte monocultures were analyzed in duplicates using multiplex assay (A; N ​= ​9), while enzyme concentrations in the supernatants were quantified in duplicates using ELISA (B; N ​= ​19). The mean concentrations and standard deviations of the cytokines GM-CSF, TNF-α, IFN-γ, IL-2, IL-4, IL-5, IL-6, IL-9, IL-10, and IL-17 are plotted (A). Enzyme concentrations of MMP-1, MMP-3, MMP-9, MMP-13, and ADAMTS-4 are plotted with mean ​± ​standard deviation (B). ∗ ​= ​P ​< ​0.05, ∗∗ ​= ​P ​< ​0.01, ∗∗∗ ​= ​P ​< ​0.001.

Enzyme and cytokine levels were quantified in chondrocyte monoculture supernatants. Among the enzymes analyzed, MMP-3 was detected at the highest concentration, significantly surpassing the others by at least 14-fold, followed by MMP-1, ADAMTS-4, MMP-13, and MMP-9 in descending order (Fig. 3A). Cytokine analysis revealed IL-6 being the most abundant cytokine, with a mean concentration of 696.2 ​pg/mL, followed by relatively lower IFN-a, IL-4, IL-2, IL-9, IL-17, IL-5, GM-CSF and TNF-a levels (Fig. 3B).

### 3.3 Comparing metalloproteinase production in Co-cultures and monocultures of chondrocytes and T cell subsets

**Fig. 4:**
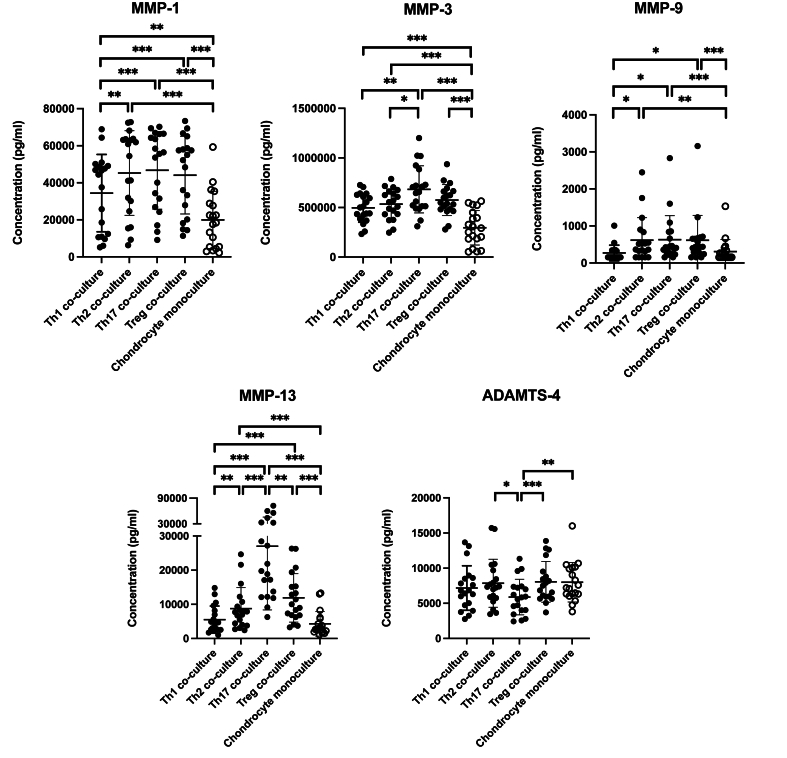
**Production of metalloproteinases in chondrocyte and T cell subset mono- and co-cultures**. Concentrations of MMPs and ADAMTS in supernatants of chondrocyte mono- and co-cultures with Th1, Th2, Th17, and Treg were quantified using ELISA. The levels of all metalloproteinases were below detection level in Th cell monocultures and therefore not statistically analyzed. Enzyme concentrations of MMP-1/-3/-9/-13 and ADAMTS-4, are plotted with mean ​± ​standard deviation (N ​= ​19). ∗ ​= ​P ​< ​0.05, ∗∗ ​= ​P ​< ​0.01, ∗∗∗ ​= ​P ​< ​0.001.

To investigate the impact of different T cell subsets in presence of chondrocytes on production of metalloproteinases, we measured MMP-1, MMP-3, MMP-9, MMP-13, ADAMTS-4, ADAMTS-5 and ADMATS-7 concentrations in the supernatants of chondrocyte co-cultures with T cell subsets and their respective monocultures (Fig. 4). All metalloproteinases levels were below detection level in Th cell monocultures and concentrations of ADAMTS-5 and ADAMTS-7 were below detection level in all cultures and thus excluded from further analysis.

In all chondrocyte co-cultures with T cell subsets, significantly higher concentrations of MMP-1, MMP-3, MMP-9, and MMP-13 were observed compared to chondrocyte monocultures, except for MMP-13 and MMP-9 in Th1 co-cultures (Fig. 4). Compared to all other Th subset co-cultures with chondrocytes, the lowest levels of metalloproteinases were generally detected in Th1 co-cultures.

Remarkably, Th17 co-cultures exhibited the highest mean concentrations for MMP-1, MMP-3, MMP-9, and MMP-13. Conversely, in Th17 co-cultures the lowest mean concentration of ADAMTS-4 were detected with no differences in ADAMTS-4 levels between chondrocyte monocultures and co-cultures with Th1, Th2 or Treg cells (Fig. 4).

### 3.4 Comparing cytokine production in Co-cultures and monocultures of chondrocytes and T-cell subsets

**Fig. 5:**
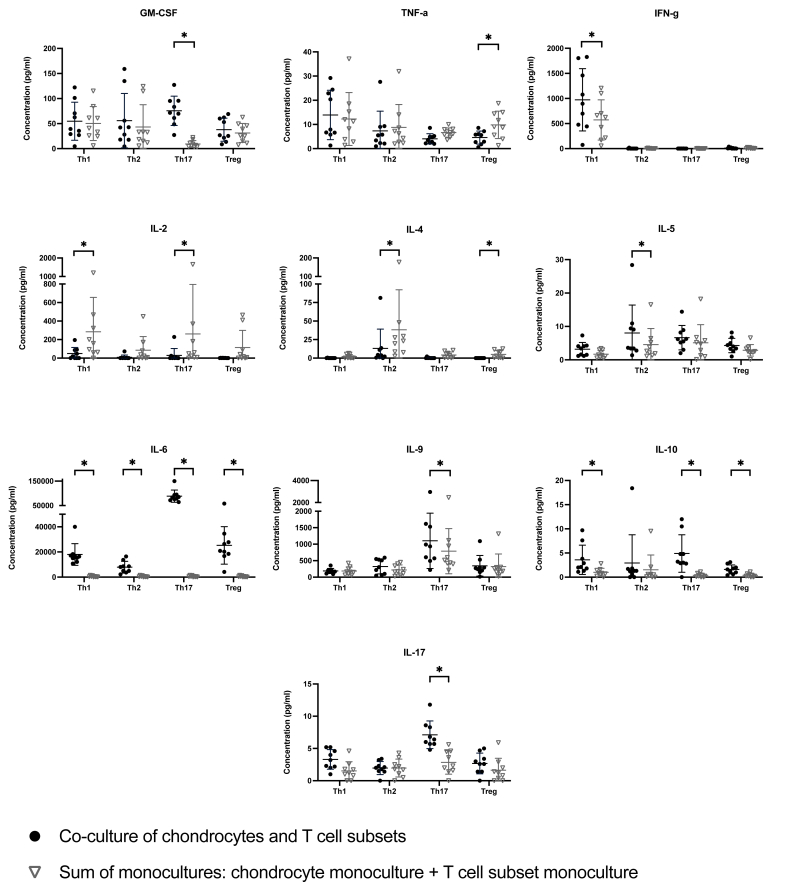
**Production of cytokines in chondrocyte and T cell subset mono- and co-cultures**. Cytokine concentrations in supernatants of chondrocyte mono- and co-cultures with Th1, Th2, Th17, and Treg were quantified using Multiplex assays (N ​= ​9). Concentrations in supernatants of co-cultures were compared with respectively monoculture approach (sum of concentrations of the chondrocyte monoculture and T cell subset monoculture). Cytokine concentrations are plotted with mean ​± ​standard deviation. ∗ ​= ​P ​< ​0.05, ∗∗ ​= ​P ​< ​0.01.

Cytokine levels were measured in supernatants of chondrocyte and T cell subset monocultures and co-cultures. To assess the impact of T cell and chondrocyte interactions, the cytokine concentrations in co-cultures supernatants were compared against the summed concentrations observed in their respective monoculture supernatants (Fig. 5).

In Th1 co-cultures, the concentration of IFN-γ was significantly elevated compared to monoculture approaches (p ​= ​0.008). IFN-γ was not detected in chondrocyte monocultures and in co-cultures with Th2, Th17 or Treg cells (Fig. 5). IL-2 levels were decreased in Th1 and Th17 co-cultures relative to their monocultures (p ​= ​0.012). Similarly, IL-4 levels were reduced specifically in Th2 and Treg co-cultures (p ​= ​0.008 and p ​= ​0.012, respectively). A substantial increase in IL-6 was observed across all co-cultures being the most pronounced in Th17 co-cultures, with an increase by almost 2000-fold compared to monoculture approaches. GM-CSF levels also rose 8-fold, while IL-9 levels increased 1.4-fold in Th17 co-cultures. Additionally, IL-17 concentration was threefold higher in Th17 co-cultures compared to monocultures. In contrast, TNF-α levels were significantly lower only in chondrocyte co-cultures with Treg cells compared to monocultures (p ​= ​0.008). Furthermore, IL-10 concentrations were elevated in Th1, Th17, and Treg co-cultures relative to monocultures (Fig. 5).

## 4 Discussion

Inflammation in OA joints disrupts cytokine balance and increases metalloproteinases (MMPs and ADAMTS) production contributing to cartilage degradation [1]. Synovial cells and chondrocytes are significantly involved in production of these enzymes and cytokines [4,6,8,9]. CD4^+^ T cells belong to the most abundant immune cell populations besides macrophages in osteoarthritic knee joints differentiating into pro-inflammatory subsets (Th1, Th17) and therefore suggested to play a significant role in OA inflammation and pathology [[13], [14], [15],^18^,^19^]. This in vitro study indicates a subset-specific interplay between CD4^+^ T cells and chondrocytes from OA patients, leading to differential modulation of cytokine and metalloproteinase production, with Th17 ​cells exerting the most pronounced inflammatory effects.

Whereas Th1 and Th17 ​cells are known for their catabolic and pro-inflammatory effects, Th2 and Treg cells are believed to possess anti-inflammatory potential [9,16,17]. CD4^+^ T cell subsets have been detected not only in synovial membrane but also in synovial fluid within OA knee joints [[18], [19], [20]]. Therefore, they may interact directly with chondrocytes exposed by disrupted cartilage matrix [11]. However, to date, the interactions between T cell subsets and chondrocytes in modulating the release of metalloproteinases and cytokines has not been explored. The aim of our study was to investigate in vitro whether chondrocytes from OA patients interact with CD4^+^ T cells and alter cytokines and metalloproteinases production and whether these changes depend on the T cell subset. By characterizing these T cell subset-specific impact, this study aimed to assess the potential for T cell targeted therapeutic interventions to address OA-related pathophysiology.

Given the absence of commercial kits tailored to our study's needs and the limited cell number of each T cell subset in synovial tissue, we developed a novel protocol for CD4^+^ T cell subset polarization from PB. This assay successfully induced and maintained Th1, Th2, Th17 and Treg subsets over a 48-h culture period. Efficacy and stability of this assay was confirmed through flow cytometry and validated against a previously published study [27], ensuring the reliability of our approach. Our results confirmed successful polarization of Th1, Th2, Th17, and Treg cells, based on their extra- and intracellular profile [27]. Th1 cells expressed CXCR3 and T-bet, Th2 cells expressed CCR4 and GATA3, Th17 ​cells showed high expression of RORγt and Treg cells were marked by CD25 and FoxP3 expression. Importantly, the polarization, survival and proliferation rates of these T cell subsets were not adversely affected by chondrocytes under the same culture conditions implying that the detected changes in cytokine and enzyme concentrations in this study are due to interactions between chondrocytes and T cell subsets. These interactions may be mediated by direct T cell-chondrocyte contact and cytokine-driven paracrine signaling [4,11,21,26,28,29]. Chondrocytes can exhibit antigen-presenting capabilities, and their secreted antigens have been identified as potential autoantigens in osteoarthritis [11]. In vitro CD3^+^ T cells proliferate strongly in the presence of OA chondrocytes. This effect can be abolished by blocking Major Histocompatibility Complex (MHC) class I and II [26] indicating MHC-mediated interactions. Moreover, metalloproteinase production was elevated in direct CD3^+^ T cells and chondrocyte co-cultures compared to transwell cultures, suggesting that cell-cell contact amplifies catabolic responses [21]. These findings indicate that observed effects in this study were mediated not only by soluble factors but also through direct contact-dependent mechanisms. However, the molecular nature of these interactions was not directly investigated and our interpretations remain speculative.

First, we analyzed mono- and co-culture supernatants of chondrocytes from OA patients with T-cell subsets regarding metalloproteinase levels. Metalloproteinases can cleave essential structures of the extracellular matrix and are key factors in cartilage degradation in OA [6]. Our findings show that CD4^+^ T cell subsets do not produce detectable levels of metalloproteinases in monoculture, consistent with a previous study [21]. Contrary, chondrocytes produced metalloproteinases to varying degrees in monoculture, with MMP-3 production being the highest. These results are consistent with a previous study and strengthen the hypothesis of cartilage-induced cartilage metabolism in OA [25]. We observed elevated levels of metalloproteinases – specifically MMP-1, MMP-3, MMP-9, and MMP-13 – in supernatants of all chondrocyte-T cell subset co-cultures compared to chondrocyte monocultures. This indicates that T cell subsets interact with chondrocytes causing changes in MMP production by enhancing metalloproteinase production, largely independent of the specific T cell subsets involved. Contrary to our assumption, based on attributed pro-inflammatory and anti-inflammatory characteristics of the T cell subsets, Th2 and Treg cells did not exhibit suppressive effects on metalloproteinase production. Instead, each T cell subset had catabolic potential by increasing MMP production in the presence of chondrocytes from OA patients as observed in vitro before with CD3^+^ T cells [21]. Interestingly, Th1 co-cultures exhibited the lowest levels of metalloproteinases, attributing Th1 cells the least chondrocatabolic potential among T cell subsets regarding to their impact on metalloproteinases production. This aligns with recent findings showing a rather suppressive effect of Th1 effector cytokine IFN-γ on metalloproteinase production by synovial fibroblasts [30]. IFN-γ can suppress metalloproteinase expression via *Signal transducer and activator of transcription* (STAT1) signaling, possibly through cyclic AMP response element-binding protein/p300 [31,32]. In contrast, Th17 co-cultures displayed the highest levels of all MMPs, particularly MMP-13, a key enzyme in cartilage degradation, underscoring a pivotal role of Th17 ​cells in OA pathophysiology [33]. These findings align with IL-17, secreted by Th17 ​cells, activating Nuclear Factor kappa-light-chain-enhancer (NF-κB) and Mitogen-Activated Protein Kinase MAPK pathways, promoting the expression of pro-inflammatory mediators [28,34].

As next step, we analyzed concentrations of different pro- and anti-inflammatory cytokines in chondrocyte and T cell subset mono- and co-culture supernatants and compared such concentrations between co-cultures and the sum of the respective monoculture approach to assess net changes in cytokine production. Cytokines are crucial in OA pathophysiology, either promoting inflammation and cartilage degradation or maintaining cartilage homeostasis through pro- and anti-inflammatory effects.

In chondrocyte monocultures concentrations of cytokines at varying extent where detected with IL-6 as the dominant cytokine, again indicating catabolic and inflammatory potential of chondrocytes in OA [4]. Interestingly, all co-cultures, showed elevated IL-6 levels, particularly pronounced in Th17 co-cultures – potentially mediated by IL-17 and the activation of NF-κB and MAPK pathways [34]. IL-6 possesses mainly pro-inflammatory properties [4], promotes Th17 ​cell differentiation and is reported to have a negative impact on cartilage metabolism by reducing the secretion of type II collagen and increasing the secretion of MMP-1 and MMP-13 via Janus kinase (JAK)/STAT pathway [9,35]. Contrary, to such overall pro-inflammatory effect of chondrocyte and T cell subset interaction, in all co-cultures also increased levels of IL-10 were detected. IL-10, an anti-inflammatory cytokine, exerts chondroprotective effects by increasing type II collagen and aggrecan secretion while decreasing MMP secretion by chondrocytes and inhibiting chondrocyte apoptosis [4]. In contrast to such rather T cell subset unspecific up- or downregulation of cytokine production, changes in IL-2/-4/-5, GM-CSF, IFN-γ and TNF-α were depended on T cell subset co-cultured with chondrocytes. Compared to monoculture approach only Th1 co-culture with chondrocytes revealed elevated concentrations of IFN-γ, hallmark cytokine of Th1 cells, known to exacerbate OA [9,36] – suggesting a potential positive feedback loop. Similarly, specifically Th17 co-cultures showed increased levels of GM-CSF, IL-9, and IL-17, being predominantly produced by Th17 ​cells, indicating a strong pro-inflammatory impact of Th17 ​cells [[37], [38], [39], [40]]. IL-9 is associated with growth and survival of T cells within the synovium and correlates with OA symptoms and poorer joint function, suggesting it as potential marker of OA activity [41,42]. Blocking GM-CSF leads to reduced synovitis, decreased cartilage damage, pain reduction and therefore a reduction in OA progression [43]. IL-17, categorized as proinflammatory cytokine, promotes OA progression by stimulating the expression of MMPs and catabolic processes by chondrocytes while simultaneously inhibiting the synthesis of proteoglycans by chondrocytes [4,22,44,45].

In contrast to this cytokine upregulation, IL-2, IL-4 and TNF-α production was downregulated by the interaction of T cells and chondrocytes depending on the T cell subset. IL-2 is associated with T cell activity and proliferation [46,47]. In this study IL-2 production in Th1 and Th17 co-cultures with chondrocytes were decreased suggesting that articular chondrocytes may suppress immune responses of particularly Th1/Th17 ​cells, as part of an anti-inflammatory mechanism as described previously [10,48]. In contrast, IL-4 production, typically associated with anti-inflammatory activities and cartilage protection by inhibiting proteoglycan degradation and metalloproteinase production [4], was suppressed by the interaction of Th2 and Treg with chondrocytes, again underscoring the inflammatory potential of chondrocytes in OA.

TNF-α, a key cytokine driving OA pathogenesis, leads to decreased secretion of proteoglycan and type II collagen while increasing the secretion of chondrodestructive enzymes [4,49]. Remarkably, in this study, when chondrocytes were co-cultured with Treg cells, TNF-α production was suppressed compared to monoculture approach, highlighting the chondroprotective potential of Treg and chondrocyte interaction in OA. Treg cells exert anti-inflammatory immunoregulatory effects through their receptor profile and secretion of anti-inflammatory cytokines as IL-10 [50], may thereby inhibiting chondrocyte inflammatory activity in OA [29]. Treg infiltration negatively correlates with knee OA pain and functional disability [17], indicating its relevance also in vivo.

To the best of our knowledge, this is the first study indicating that chondrocytes from OA patients can interact with CD4^+^ T cell subsets promoting changes in production of MMPs and pro- and anti-inflammatory cytokines depending on CD4^+^ T cell subset. Among T cell subsets analyzed, Th17 ​cells and its interaction with chondrocytes revealed the strongest pro-inflammatory and chondrocatabolic potential. Consequently, our study suggests, that targeting Th17 in osteoarthritic knee joints may represent a potentially effective strategy to reduce cartilage damage and slow OA progression. The interaction of chondrocytes with Th2 and Treg cells showed both destructive and protective effects, emphasizing the complexity of their role in OA. However, the increase of IL-10 production and specifically the reduction of TNF-α production of Treg in presence of chondrocytes suggest a high potential of such interaction in inhibiting cartilage breakdown.

### 4.1 Limitations

Despite the controlled experimental conditions, the findings of this study must be interpreted considering limitations inherent to in vitro models. The OA joint represents a highly complex microenvironment involving dynamic interactions between chondrocytes, synovial fibroblasts, immune cells, the extracellular matrix and mechanical load. Our co-culture system isolates the interaction between polarized CD4^+^ T cells and chondrocytes with cell numbers and culture conditions not fully replicating the complexity of OA pathophysiology. Furthermore, we did not investigate underlying molecular mechanisms and signaling pathways. The relatively small sample size limits the generalizability of our findings. Future studies should expand the study population, validate our findings and suggestions using more integrative ex vivo systems or animal models and include molecular pathway analyses.

## Author’s contributions

Conception and design: H. Platzer, B. Moradi.

Collection and assembly of data: H. Platzer, B. Moradi, M. Wellbrock, S. Maciej, B. Khamees.

Analysis and interpretation of data: H. Platzer, B. Moradi, M. Wellbrock, R. Mayakrishnan, S. Gantz.

Drafting of the article: H. Platzer, B. Moradi, M. Wellbrock, R. Mayakrishnan, G.Pourbozorg, S. Gantz.

Critical Revision of the article: H. Platzer, B. Moradi, S. Maciej, B. Khamees.

Final approval of the article: H. Platzer, B. Moradi, M. Wellbrock, R. Mayakrishnan, G.Pourbozorg, S. Gantz, S. Maciej, B. Khamees.

Responsibility for the integrity of the work as a whole, from inception to finished article:

H. Platzer, B. Moradi.

## Ethics statement

The study was approved by the Ethics Committee of the Medical Faculty of the University of Kiel (D405/22) and the procedures followed were in accordance with the ethical standards of the responsible committee on human experimentation (institutional and national) and with the Helsinki Declaration of 1975, as revised in 2000. All patients provided informed consent prior to participation.

## Role of the funding source

This study was financially supported by the 10.13039/501100001659German Research Foundation (Deutsche Forschungsgemeinschaft) and 10.13039/100018842Deutsche Arthrose-Hilfe e.V.. The sponsors had no role in study design; in the collection, analysis, and interpretation of data; in the writing of the report; and in the decision to submit the paper for publication.

## Declaration of competing interest

The authors declare that they have no conflicts of interest.

## Abbreviations

ADAMTS: a disintegrin and metalloprotease with thrombospondin motifs
CCR: C–C Chemokine Receptor
CRP: C-reactive protein
CXCR: C-X-C-Chemokin-Rezeptor
DAMPs: damage-associated molecular patterns
DMEM: Dulbecco's Modified Eagle Medium
EDTA: ethylenediaminetetraacetic acid
ELISA: enzyme-linked immunosorbent assay
FCS: fetal calf serum
FoxP3: forkhead-Box-Protein P3
FSC: Forward Scatter
GATA3: GATA binding protein 3
GM-CSF: Granulocyte-macrophage colony-stimulating factor
IFN- γ: Interferon-gamma
IL: interleukin
JAK: Janus kinase
K&L: Kellgren-Lawrence
MAPK: Mitogen-Activated Protein Kinase
MHC: Major Histocompatibility Complex
NF-κB: Nuclear Factor kappa-light-chain-enhancer of activated B cells
OA: osteoarthritis
PB: peripheral blood
PBMC: peripheral blood mononuclear cell
PBS: Phosphate-Buffered Saline
RORyt: RAR-related orphan receptor gamma
SSC: Side Scatter
STAT: Signal transducer and activator of transcription
T-bet: t-box-expressed in t-cells
Th: T helper
TNF: Tumor necrosis factor
Tregs: T regulatory cells
WBC: white blood cells
